# Engineered stem cell niche matrices for rotator cuff tendon regenerative engineering

**DOI:** 10.1371/journal.pone.0174789

**Published:** 2017-04-03

**Authors:** M. Sean Peach, Daisy M. Ramos, Roshan James, Nicole L. Morozowich, Augustus D. Mazzocca, Steven B. Doty, Harry R. Allcock, Sangamesh G. Kumbar, Cato T. Laurencin

**Affiliations:** 1 Institute for Regenerative Engineering, University of Connecticut Health Center, Farmington, Connecticut, United States of America; 2 Raymond and Beverly Sackler Center for Biomedical, Biological, Physical and Engineering Sciences, Farmington, Connecticut, United States of America; 3 Department of Orthopaedic Surgery, University of Connecticut Health Center, Farmington, Connecticut, United States of America; 4 Department of Materials Science & Engineering, University of Connecticut, Storrs, Connecticut, United States of America; 5 Department of Chemistry, the Pennsylvania State University, University Park, Pennsylvania, United States of America; 6 Analytical Microscopy Laboratory, Hospital for Special Surgery, New York, NY, United States of America; University of California, San Diego, UNITED STATES

## Abstract

Rotator cuff (RC) tears represent a large proportion of musculoskeletal injuries attended to at the clinic and thereby make RC repair surgeries one of the most widely performed musculoskeletal procedures. Despite the high incidence rate of RC tears, operative treatments have provided minimal functional gains and suffer from high re-tear rates. The hypocellular nature of tendon tissue poses a limited capacity for regeneration. In recent years, great strides have been made in the area of tendonogenesis and differentiation towards tendon cells due to a greater understanding of the tendon stem cell niche, development of advanced materials, improved scaffold fabrication techniques, and delineation of the phenotype development process. Though *in vitro* models for tendonogenesis have shown promising results, *in vivo* models have been less successful. The present work investigates structured matrices mimicking the tendon microenvironment as cell delivery vehicles in a rat RC tear model. RC injuries augmented with a matrix delivering rat mesenchymal stem cells (rMSCs) showed enhanced regeneration over suture repair alone or repair with augmentation, at 6 and 12-weeks post-surgery. The local delivery of rMSCs led to increased mechanical properties and improved tissue morphology. We hypothesize that the mesenchymal stem cells function to modulate the local immune and bioactivity environment through autocrine/paracrine and/or cell homing mechanisms. This study provides evidence for improved tendon healing with biomimetic matrices and delivered MSCs with the potential for translation to larger, clinical animal models. The enhanced regenerative healing response with stem cell delivering biomimetic matrices may represent a new treatment paradigm for massive RC tendon tears.

## Introduction

Tendon injuries constitute a significant unmet clinical need with rotator cuff (RC) pathology being highly prevalent [1] and mostly involving tears of the supraspinatus tendon in the shoulder [2]. This musculotendon unit is solely responsible for the first 30 degrees of arm abduction, and an injury presents considerable morbidity [3]. Unfortunately, most massive RC tendon injuries suffer from re-tears and require post-procedure surgical intervention to reestablish tissue continuity. We have optimized biomaterial based fiber matrices to mimic the native extracellular architecture of tendon tissue via properties such as material stiffness, fiber organization, and the presentation of cues [4]. However, *in vivo* augmentation with a biomimetic matrix alone may not suffice to instruct the host cells to remodel and enhance regeneration of hypocellular tissues such as tendons and ligaments. We must converge our deep and better understanding of developmental biology, biological chemistry, and molecular level interactions that govern cellular behavior [5,6] to direct stem cells to emulate the process of tissue development, differentiation, and growth of complete multi-cellular tissues such as the limb [7]. Laurencin *et al*. defined this new field as ‘Regenerative Engineering’, which is the convergence of advanced materials science, stem cell science, physics, developmental biology, and clinical translation to answer the grand challenge, i.e. regenerate complex multi-cellular tissues [6].

Stem cells due to their proven pluripotent nature and self-renewal capacity are a natural choice for the development of cell-based tissue engineering strategies [8–10]. It is today widely recognized the presence of stem cells in almost all adult tissues [11,12] and they exhibit superior powers such as the ability for self-renewal, growth, and differentiation into a variety of cell phenotypes [13–15]. The conventional *in vitro* cell expansion approach where cells are grown on smooth and stiff tissue culture plastic under media conditions and lacking necessary stimulatory cues is challenging [16], with cells undergoing phenotypic drift and senescence leading to poor clinical translation of promising therapies [17]. The tissue microenvironment sustains adult stem cells by maintaining a balance between the states of quiescence, self-renewal, and differentiative capacity [14,18–21]. Engineered scaffolds aim to replicate the instructive microenvironment, and is crucial to recapitulating tissue architecture, physiochemical properties, and the signaling pathways which support cross communication of the state and requirements of the tissue with cells by means of cues [22,23]. The diversity and dynamic remodeling nature of the extracellular matrix (ECM) provide for cellular environments driven by cues such as biochemical, physical, structural and mechanical stimuli [24,25]. The stem cell–ECM interaction is largely a feedback relationship represented by reciprocity in stem cell behavior and ECM remodeling [26,27].

Tendons are assembled of densely packed collagen fibers that exhibit a hierarchically increasing collagen bundle organization. Amid the current regenerative technologies, electrospun fiber matrices show promise for tendon healing and repair due to the biomimetic nature of non-braided matrices to the native tendon ECM [4]. We created a hybrid polymer fiber matrix by first electrospinning polycaprolactone (PCL) mimicking the structural organization and cellular microenvironment of the rotator cuff tendon tissue, and then surface functionalizing the tendon microenvironment-like matrices with polyphosphazene poly[(ethyl alanato)_1_(*p*-methyl phenoxy)_1_] phosphazene (PNEA-mPh) to improve matrix hydrophilicity [28]. *In vitro* studies demonstrated that mimicking the tendon microenvironment and increased hydrophilicity by surface functionalization improved initial mesenchymal stem cell adhesion, long-term cell permeation, and promoted tendonogenic differentiation [29].

Bone-derived mesenchymal stem cells (MSCs) are multipotent, self-renew, and lack histo-incompatibility, and provide exogenous regenerative cues during RC repair [30–32]. Further, bone-derived MSCs are easily acquired during anchor hole placement in human arthroscopic rotator cuff repair [33]. Animal studies involving non-rotator cuff tendons have shown the potential of cell seeding in improving tendon repair [34,35]. For example, cell delivery to a torn Achilles tendon resulted in greater strength and more native-tissue like histology [34]. Human studies have demonstrated improved functionality when bone-derived MSCs are applied to massive RC tear repair [35]. However, little is known in regards to a combinatorial treatment strategy using a biomimetic scaffold for augmentation and for the delivery of an exogenous stem cell population for repair of massive tears of the RC [36]. The hybrid PCL/PNEA-mPh electrospun matrix mimics the tendon tissue microenvironment, functioning as a delivery vehicle for rat MSCs, and augments the repair in a rat model of RC laceration. While the applied MSCs did not incorporate into the regenerating tissue, their delivery improved mechanical characteristics and tissue composition in a way that was unachievable with the scaffold alone augmentation. The MSCs delivered using a biomimetic hybrid scaffold may exhibit autocrine/paracrine modulation, and in light of the chemotactic effect may be enhancing regeneration via stem cell homing. This is a proof-of-concept study demonstrating that MSCs delivered via a tendon microenvironment-mimicking scaffold improves tendon healing.

## Materials and methods

### 1. Fabrication of PCL/PNEA-mPh fiber matrices

Design, synthesis, and characterization of PNEA-mPh, PCL and PNEA-mPh modified fiber matrices via electrospinning has been published elsewhere [28]. Briefly, PCL was dissolved in solvent admixture of MeCl:EtOH::85:15 at a concentration of 17.5%, and electrospun using 18G blunt tip needles under optimized conditions of constant 4 mL/h flow rate, 1 kV/cm electric potential, ambient temperature, and humidity. The electrospun PCL fiber matrices of 5 mm × 7 mm were functionalized by dip-coating in 2% PNEA-mPh solution prepared in a solvent admixture of AcO:EtOH (50:50) for one second at room temperature and allowed to air dry for 15 minutes. Coated fiber matrices were further dried for 24 h under vacuum and desiccated until further use.

### 2. *In vivo* rc injury, suture repair and integrative augmentation model

**Fig 1:**
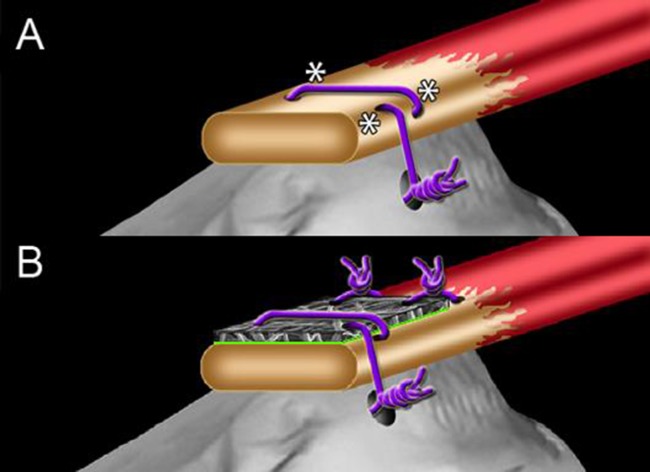
Non-augmented and augmented rat supraspinatus repair model (***A***) Modified MasonAllen stitch described by Soslowsky. Purple indicates suture, * indicates areas of stress. (***B***) Integrated matrix augmentation model for supraspinatus tendon repair. Green indicates the side of cell seeding in matrix/rMSC group.

This animal study was carried out in accordance with the recommendations in the Guide for the Care and Use of Laboratory Animals of the National Institutes of Health. The protocol was approved by the Institutional Animal Care and Use Committee (IACUC) of the University of Connecticut Health Center (PHS Assurance No.: A3471-01). All surgery was performed under inhalational isoflurane anesthesia and all efforts were made to minimize suffering. Surgeries were performed following standard aseptic technique procedures on male (retired breeder) Sprague Dawley rats (450 g, Harlan Laboratories, Indianapolis, IN). The rats were housed individually with ad libitum access to food and water. Repair (R) was performed using the Modified Mason-Allen stitch described by the Soslowsky group (57), while RC augmentation (R+S) involved few modifications. Namely, a 5 mm x 7 mm matrix was placed on top of the tendon at the repair site after the initial pass of a 6–0 Prolene double-armed suture. The matrix was trimmed to 3 mm x 3 mm and the remainder of the Modified Mason-Allen stitch was performed, securing the distal portion of the matrix to the humerus. The proximal portion of the matrix was fixed to the tendon with two loops of 6–0 Prolene resulting in repair augmentation as shown in **Fig 1**. In the third experimental group, matrices seeded with MSCs (R+S+C) were used to augment and deliver cells to the RC tendon injury. Animals were operated on and allowed to heal for the indicated time points and numbers as detailed in **Table 1**.

**Table 1:** Outline of Animal Studies.

Assessment	# Rats	Time Points (Weeks)	Procedures Performed	Rat Total
Cell Tracing	3	6, 12	R+S+C	6
Tensile Properties	7	6, 12	R, R+S, R+S+C	42
Histology	3	6, 12	R, R+S, R+S+C	18
**Grand Total Rats Used**				**66**

Key: R = Repair, R+S = Matrix Augmentation, R+S+C = Matrix/rMSC cell delivery

### 3. Rat MSC culture and matrix seeding

Sprague-Dawley bone-derived MSCs were expanded until passage 4 on 150 mm dishes incubated at 37°C and 5% carbon dioxide. Cultures were fed every other day with basal media consisting of alpha-MEM (without phenol red) (Invitrogen, Carlsbad, CA) supplemented with 10% FBS and 0.1% P/S. PCL/PNEA-mPh matrices were sterilized by incubating in 70% ethanol solution for 20 minutes and then exposed to UV for 30 minutes on each side in a cell culture hood as previously described in Peach *et al*. [29]. Under sterile conditions, these constructs were transferred to sterile 96 well plate and preconditioned in growth media for 3 h. Seeding took place at a density of 30K cells in 50 μL of media per construct at 37°C. After 3 h the culture volume was brought up 250 μL per well and was exchanged after 24 h.

### 4. MSCs delivery to RC

Constructs were implanted after 48 h post-seeding in the culture medium. Previously reported cell attachment studies demonstrated good cell adhesion over a period of 2 days. Constructs were applied to the integrated augmentation model as described above with care taken to quickly but gently place the construct on top of the tendon with the cell seeded side facing the tendon. During the remainder of the surgery, the scaffold was frequently dabbed with tissue fluids to prevent construct desiccation.

### 5. Tissue harvesting, fixing and processing

Rats were euthanized, and the RC tendon–humerus bone tissue was immediately dissected and fixed as detailed elsewhere [29] with a 3x wash in PBS followed by 4°C incubation in 4% paraformaldehyde (PFA) (pH 7.4). Samples undergoing frozen histology were fixed for 24 hours, while the rest were allowed to incubate for 72 hours. Samples were then transferred to 70% EtOH at 4°C briefly before further processing. Samples (RC tendon-humerus bone tissue) were decalcified through immersion in 5% EDTA for one week at 4°C, then washed and dehydrated for 10 minutes serially in 70%, 95% and 100% EtOH. The samples were embedded in a low melting point embedding wax (Tissue-Tek VIP, Sakura Finetek USA, Inc., Torrance, CA), which did not surpass PCL’s melting point of 60°C so the polymer matrices remained intact. Samples were sectioned at 5 μm, baked, and deparaffinized by sequential incubation for 10 minutes in the following solutions: xylene, 1:1 xylene:EtOH, 100% EtOH, 95% EtOH, 70% EtOH, 50% EtOH and DI water.

### 6. Histology staining

Standard stains were applied to decalcified sections using typical protocols, and images were acquired at 20x and 40x magnification. Hematoxylin and eosin (H&E) stain was applied to decalcified sections to assess overall morphology, while Masson’s trichrome stain was employed to better distinguish tendon connective tissue from the surrounding muscle and bone.

### 7. Collagen immunohistochemistry- histological semi-quantitation

The ratio of Col I: Col III coverage on the tendons at 12-weeks post-injury was determined for all four groups using semi-quantitative histology. Monoclonal Col I (Cat. # ab6308, Abcam, MA) and Col III (Cat. # ab6310, Abcam, MA) antibodies were incubated with deparaffinized samples at a 1:2000 dilution overnight (37°C) and then imaged using a DAB peroxidase-based detection system. Evenly spaced 20x fields of the tendon in the control and the suture repaired tendon, as well as in the tendon underlying the two different augmentation groups were imaged at the same level of exposure and light intensity using an Olympus BX51. To subtract counter H&E staining in the produced image files the color deconvolution plug-in was applied to the images in ImageJ such that only the DAB stain was in view [37]. The color of the resulting images was desaturated and the same level grayscale threshold was applied to each image from which the area of staining was calculated using ImageJ. The Col I: Col III ratio of coverage for each rat was obtained by taken the average of the ratio of these fields.

### 8. Quantification of collagen organization-polarized microscopy

Paraffin-embedded samples were stained with Picrosirius and observed under an Olympus BX51 scope with double polarized filters. Three evenly spaced 20x objective views of the tissue underlying the augmentation scaffold, and across intact and repaired tendon was observed. The analyzer polarized filter was rotated until the greatest intensity of light transmission was reached for each location, at which point images were acquired. The red channel of the resulting images, which isolates the birefringent signal, was averaged in ImageJ to get a grayscale value, which is equivalent to the average birefringence signal for that field. Using 8-bit image output, values were applied to a scale having theoretical values from 0–256 the level of birefringence, permitting tissue birefringence to be quantified and compared between the 4 experimental groups.

### 9. rMSCs staining with plasma membrane cell tracer dye

For the purpose of tracing donor cells in the rat augmentation animal model rMSCs were stained with the PKH26 plasma membrane dye (half-life– 100 days in red blood cells) as reported in the literature [38]. Cells were plated at a density of 1 million cells per 150 mm plate. Once 90% confluent, these cells were used for construct seeding as described in Peach *et al*. [29].

### 10. Frozen histology for fluorescent dye cell tracing

Fixed samples were embedded immersed in liquid nitrogen with Tissue-Tek* CRYO-OCT and sectioned using the Cryojane system installed into a Leica CM3050 S cryostat (Lecia Microsystems, Richmond, IL) set at 20 μm. The slides were mounted with glycerol and imaged by DIC (20x magnification) to obtain images of overall tissue and matrix morphology and visualization for red fluorescence to ascertain the presence of dye traced donor cells.

### 11. Tissue sample preparation and potting for mechanical testing

Samples were given a numerical label and blindly dissected so that all that remained was the humerus and the supraspinatus tendon. The humerus was mounted in 11 mm diameter stainless steel collars using orthopaedic resin, using a 2:1 ratio of liquid to powder components. Once set after 30 minutes the average cross-sectional area of the potted tendon was measured using a digital caliper and digital thickness gauge.

### 12. Tensile testing apparatus and testing procedure

The lower clamp of the Instron was equipped with a custom fabricated clamp that was able to tighten around a 11 mm outside diameter steel collar, allowing the collar to rotate and move in all 3 axes. This allowed proper alignment of the tendon, which was secured in a serrated clamp on the top fixation head. A macro lens attached to a digital SLR (Canon T1i) with 1080p 30 fps video capabilities was positioned such that the exposed areas of samples were able to be video recorded at high resolution. With the samples placed in the clamp, phosphate buffered saline was periodically applied with cotton swabs to prevent tissue desiccation. A 9–0 suture dipped in permanent ink was used to apply dye lines across the tendon perpendicular to the strain axis. A load of 0.1 N was applied for 60 seconds, followed by a relaxation period for 60 seconds, and then a strain rate of 6 mm/min was applied until failure. The movement of the lines marked on the tendon was recorded by the DSLR camera and used as the real extension and strain of the tissue, which was graphed against the applied Instron load. Elastic modulus was determined from the slope of the stress- strain curve.

### 13. Statistical analysis

Statistical evaluations were based on analysis of variation (ANOVA) followed by Tukey’s HSD (Honestly Significant Differences) analysis of the differences between groups with a confidence range of at least 95%. GraphPad Prism statistical software was used to determine differences between the experimental groups, the standard deviation, and to plot the graphs.

## Results

### 1. Characterization of repaired tendon tissue organization

**Fig 2:**
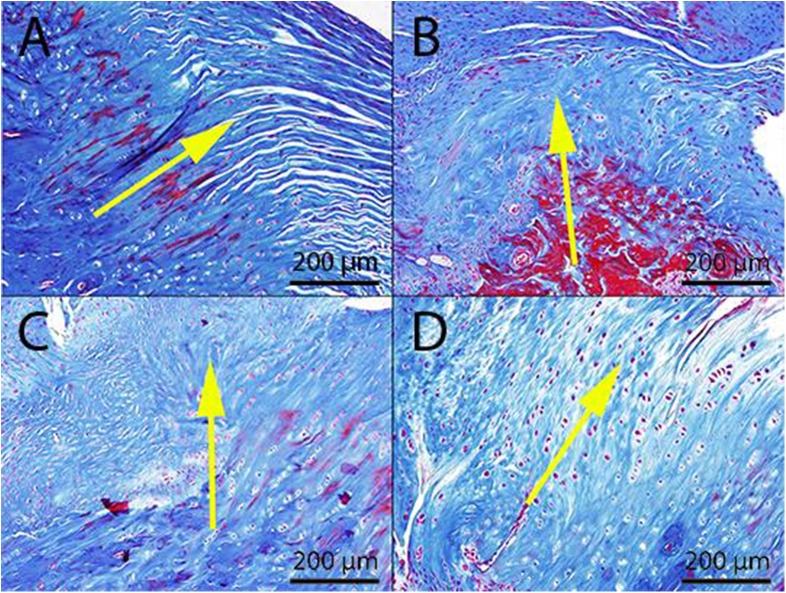
Insertion morphology of repair, matrix augmentation and matrix/rMSC repair of rat supraspinatus tendons. Native (**CON**) tendons (***A***) demonstrate a gradual transition from parallel oriented collagenous tendon tissue to bone with a cartilage intermediate. Repair (**R**) (***B***) and matrix augmented (**R+S**) (***C***) insertions have an abrupt transition. Matrix/rMSC repair (**R+S+C**) (***D***) insertions demonstrate a transition and organization similar to the intact tendon. Representative samples at 12-weeks post-surgery stained with Masson’s trichrome. Matrix/sutures are located on the surface plane above the tendon-bone insertion and are present at both 6 (not shown) and 12-weeks. Yellow arrow indicates the bone-tendon axis. Scale bars 200 μm.

Tendon reconstruction of the suture only repair (**Fig 1*A*)** and the augmentation groups (**Fig 1*B***) remained intact at 6 and 12-week time points. All rats were able to bear weight and acquired the full range of shoulder motion by 7 days post-surgery, and no animals were lost during or subsequent to surgery. Intact supraspinatus insertions exhibit tendon tissue with parallel oriented fibers (**Fig 2*A***) that blend into a gradual cartilaginous transition before reaching the bone. A representative insertion of a suture only repair at 12-weeks shows disorganized collagen in the tendon with an abrupt transition to bone and little cartilaginous material within the transition zone (**Fig 2*B***). While similar observations were made for the scaffold alone group (**Fig 2*C*)**, the rMSCs delivery group took on more of an intact tendon appearance. As shown in the 12-week example in **Fig 2*D***, there is a gradual transition from tendon to bone with the tendon insertion organized in parallel arrangements. In total 1/3 rats and 2/3 rats at 6 and 12-weeks respectively attained more native insertion morphology.

**Fig 3:**
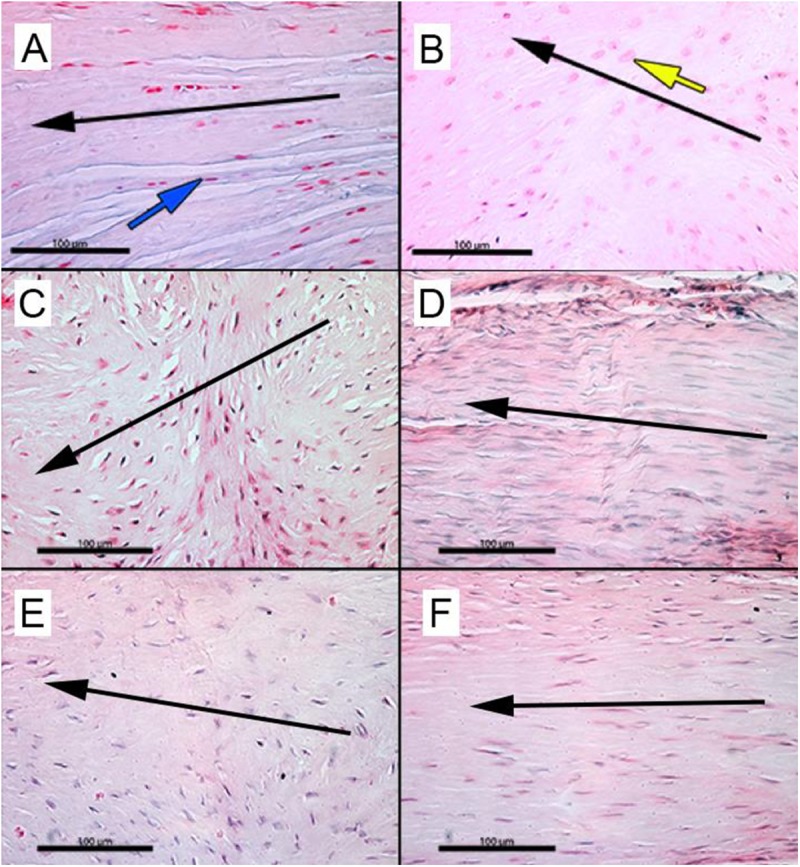
Representative morphology of intact, repaired, and augmented supraspinatus tendon mid-substance. Representative slide of native (**CON**) tendon demonstrates a matrix with low cellularity, with a flattened appearance (**blue arrow**) aligned to parallel collagen fibers (***A***). In contrast, repaired (**R**) tendons up to 12-weeks demonstrate a disoriented collagen matrix with a hypercellular population of larger, rounded, non-oriented cells (**yellow arrow**) (***B***). Disorganized matrix was observed in the tendon body underlying matrix augmentation (**R+S**) at 6-weeks (***C***) and 12-weeks (***E***) while matrix/rMSC (**R+S+C**) group results in fiber orientation at both time points (6-weeks (***D*)**, 12-weeks (***F*)**). Black arrows indicate anatomical stress axis. Scale Bars 100 μm.

Morphology of the tendon mid-substance indicated additional differences between experimental groups. Native tendon (CON), as stained with H&E, possessed parallel collagen fibers with low cellular (tenocyte) density (**Fig 3*A***), whereas a representative 12-week suture repair shows a large number of rounded cells dispersed in a disorganized matrix (arrow, **Fig 3*B***). Similarly, matrix disorganization intermixed with a high cellular density of rounded cells was observed in the tendon mid-substance underlying scaffold augmentation at both time points (**Fig 3*C* and 3*E***). The delivery of MSCs preserved fiber organization within the tissue, and this tendon tissue is composed of cells that are elongated and qualitatively has a lower cell number compared to scaffold alone augmentation at 12-weeks post-procedure (**Fig 3*D* and 3*F***).

### 2. Col I: Col III ratio of repaired RC tendon tissue

**Fig 4:**
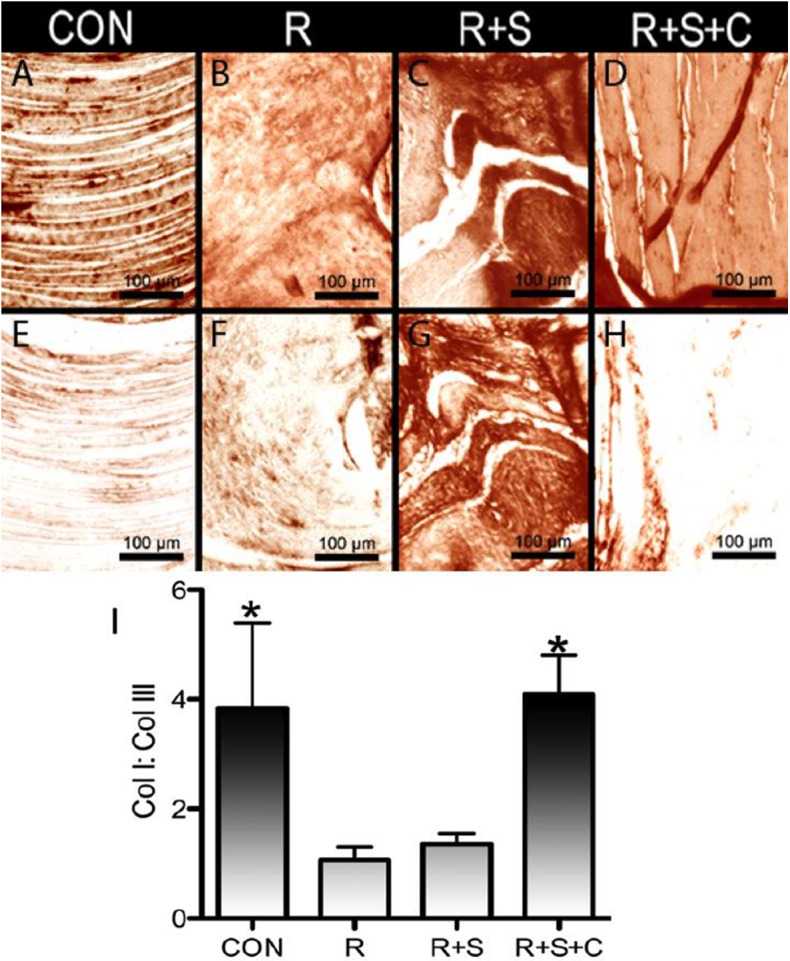
Col I and Col III expression during supraspinatus repair and augmentation. Col I (***A*, *B*, *C*, *D***) and Col III (***E*, *F*, *G*, *H***) signal was observed from immunohistology staining of native supraspinatus tendon (**CON**) and tendon tissue harvested at 12-weeks after repair (**R**), matrix augmentation (**R+S**) and matrix/rMSC (**R+S+C**). Both native tendon and tendon underlying matrix/rMSC group demonstrate a small area of Col III staining. Semi-quantification of the Col I: Col III area of expression (***I***). Both intact tendon and tendon underlying matrix/rMSC group demonstrate a larger ratio of Col I: Col III expression. n = 3 animals per group, * = p<0.05. Scale bars 100 μm.

Tendon healing is strongly correlated to the ratio of Col I and Col III expression within the tendon tissue [39,40]. We used immunohistological semi-quantification to determine the ratio of area expressing Col I and Col III for the tendon mid-substance in control and suture repair, as well as in the tendon mid-substance underlying the scaffold alone and rMSCs/augmentation groups. As demonstrated in **Fig 4*A* and 4*E***, the intact tendon has a small fraction of Col III staining, resulting in a large Col I: Col III area expression ratio (**Fig 4*I***). Conversely, the matrix of suture repaired tendons has a significant expression of Col III, leading to a ratio of approximately 1:1 (**Fig 4*B*, 4*F* and 4*I***). In line with the aforementioned morphological assessments, tendon underlying rMSCs delivery groups has a staining pattern and ratio similar to the intact tendon (**Fig 4*D*, 4*H* and 4*I***). Scaffold augmentation alone led to tendon tissue possessing a greater fraction of Col III area staining similar to the level observed for suture repaired tendon (**Fig 4*C*, 4*G* and 4*I***). The ratio of Col I: Col III within the repair tissue is similar to that of intact RC tendon as would be expected for a significantly improved healing response (**Fig 4**).

### 3. Characterization of tissue maturation

**Fig 5:**
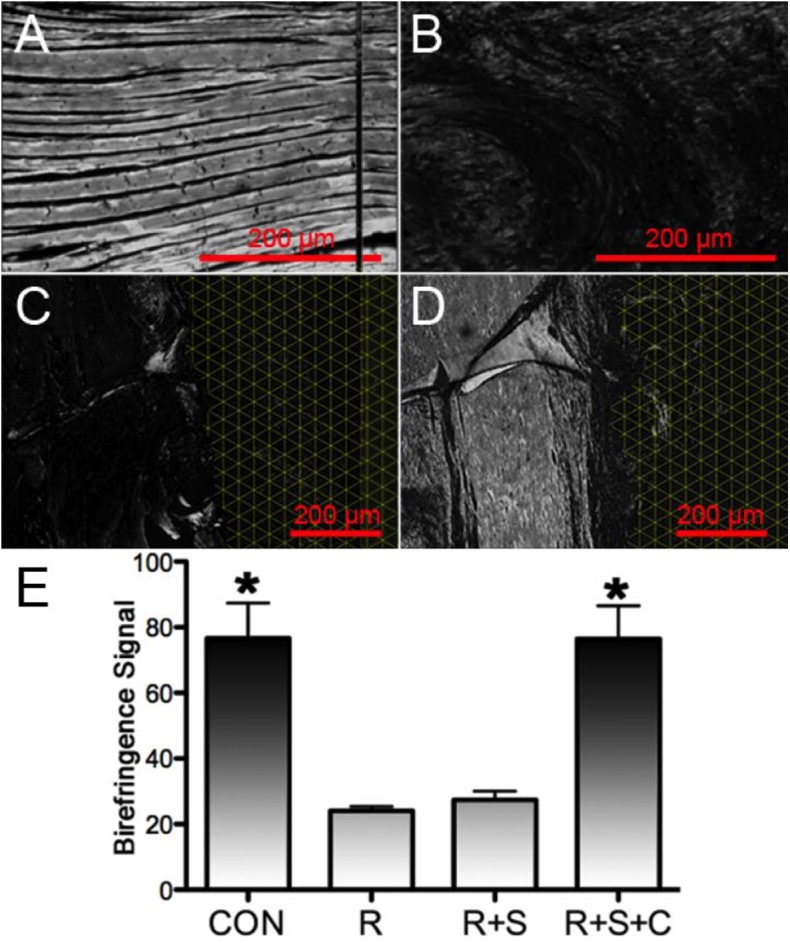
Imaging and quantification of collagen organization during supraspinatus repair and augmentation. Slides from native tendon (**CON**) (***A***) and tendons harvested 12-weeks after repair (**R**) (***B***), matrix augmentation (**R+S**) (***C***) and matrix/rMSC (**R+S+C**) (***D***) were stained with picrosirius red and observed under cross-polarized light. Both intact tendon and tendon underlying matrix/rMSC repair demonstrate a high level of birefringence that highlights tissue with highly oriented collagen fiber morphology. Average birefringent signaling from cross-polarized light microscopy of picrosirius red stained slides was converted to an 8-bit grayscale to quantitatively compare the degree of collagen orientation in tendon tissue (***E***) from native supraspinatus tendon (**CON**) and tendons harvested 12-weeks after repair (**R**), matrix augmented repair (**R+S**) and cell seeded augmented repair (**R+S+C**). Both intact tendon and tendon underlying cell seeded augmented repair demonstrate a significantly greater collagen orientation. n = 3 animals per group, * = p<0.05. Scale bars 200 μm, matrix above tendon marked by the yellow grid.

Tissue birefringence has been shown to correlate with collagen fiber bundle orientation and the degree of tendon healing in several RC studies [41–44]. Using 8-bit grayscale images, tissue birefringent transmittance could be quantitatively compared between groups by averaging the brightness over microscope fields and applying it to a 0–256 scale of brightness. With such quantification, the birefringence of tissue with theoretically no collagen orientation would have a value near 0, while perfectly aligned collagen tissue permitting 100% transmittance of double polarized light would have a value approaching 256. The observations between the experiments groups in **Fig 5*A*, 5*B*, 5*C* and 5*D***translated into a quantifiable pattern of collagen fiber organization in which the tendon tissue underlying the rMSCs delivery group has an analogous level of cross-polarized light transmittance as intact tendon, and both having significantly greater transmittance than that determined for repair and scaffold augmentation (**Fig 5*E***). Suture alone repair and matrix augmentation groups in the present work (**Fig 5**) demonstrated less birefringence transmission from the tendon body as compared to the augmentation and cell delivery repair group and intact tendon.

### 4. Characterization of mechanical properties of intact, repaired and augmented rat supraspinatus tendons

**Fig 6:**
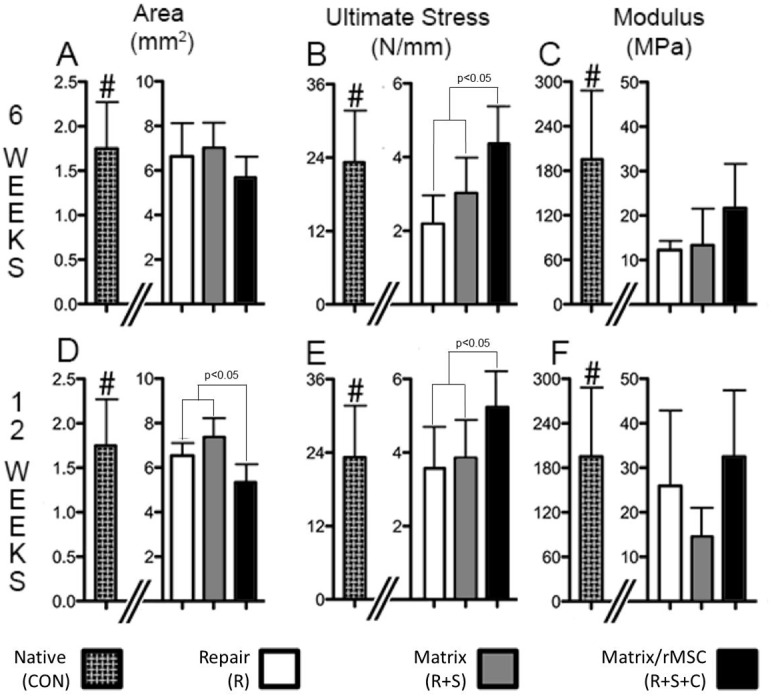
Cross-sectional area (A-6-weeks, D-12-weeks), ultimate stress (B-6-weeks, E-12-weeks) and modulus (C-6-weeks, F-12-weeks) of native (CON), repaired (R), augmented supraspinatus (R+S), and matrix/rMSC (R+S+C) tendons at 6 and 12-week post-surgery. Native tendons possessed significantly less cross-sectional area, greater ultimate stress and greater tensile modulus than all experimental groups at both time points. At 12-weeks the cross-sectional area of matrix/rMSC (R+S+C) tendon was less than the other experimental groups. There was no significant difference in cross sectional area between 6 and 12-week specimens within each experimental group. The ultimate stress (B and E) of matrix/rMSC (R+S+C) tendons was significantly greater than suture repair (R) and augmented repair (R+S) at 6 and 12-weeks. There was no significant difference in ultimate stress between 6 and 12-week specimens within each experimental group with the exception of the repair group (R). The tensile modulus (C and F) of matrix/rMSC tendon was significantly greater than suture repair (R) at 6-weeks and showed a trend of greater modulus than augmented repair (R+S) at 12-weeks. There was no significant difference in tendon modulus between 6 and 12-week specimens within each experimental group with the exception of a trend of increased stiffness for matrix/rMSC. # = p<0.05 (vs. all other groups)

As shown in **Fig 6**, mechanical characteristics of the intact tendon in regards to modulus, ultimate load, and ultimate stress are in line with previous reports [45–48]. Cross-sectional area, ultimate stress, and modulus of the repaired RC tendon in the experimental groups deviated sharply from intact tendon as expected from studies of rat supraspinatus repair [48] with and without matrix augmentation or biologic factors [49]. As compared to the repaired tendon groups, the native tendon has a significantly smaller cross-sectional area (**Fig 6*A* and 6*D***). All groups had similar measurements at 6 and 12-weeks with the exception of a small decrease in cross-sectional area of the rMSCs delivery group relative to the other experimental groups. Using a strain of 6 mm/min with a 0.1N 60-second preload, native supraspinatus tendons possessed significantly greater tendon mechanical properties over experimental groups, with a tensile modulus and ultimate stress of 195.1 N/m^2^ and 23.2 N/m, respectively (**Fig 6*B*, 6*C*, 6*E* and 6*F***). Among the experimental groups, the rMSCs delivery group demonstrated a trend of greater modulus at both time points, with greater modulus at 6-weeks over suture repair and at 12-weeks over scaffold augmentation (**Fig C and F**). Ultimate stress established clearer relationships between the experimental groups as shown in **Fig 6*B* and 6*E***. At both 6 and 12-weeks, the delivery of rMSCs resulted in significantly greater ultimate stress over suture repair and scaffold augmentation. In comparison to the augmentation groups which had a modest temporal increase in ultimate stress, only the suture repair group showed a significant increase from 6 to 12-weeks. A similar deviation is observed in primate studies in which repair only achieves 60% of the modulus one-year post suture repair [50].

### 5. Fate of exogenous MSCs in the engineered niche

**Fig 7:**
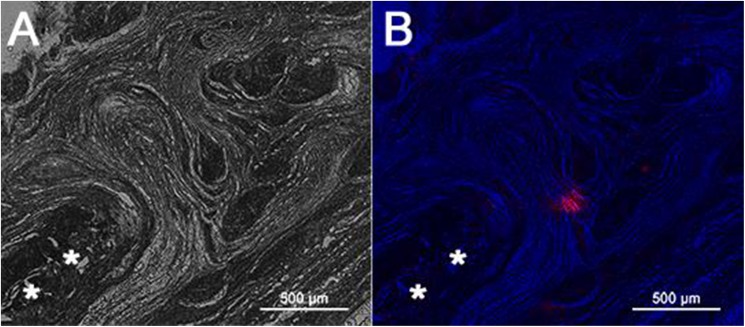
Tracing of donor rMSCs during supraspinatus augmentation. Rats underwent repair with matrices seeded with rMSC stained with PKH26 plasma membrane dye (half-life = 100days). The matrix (**White Asterisks**) was observed on unstained slides under differential interference contrast microscopy (***A***). Only one of three rats at 6-weeks post-surgery demonstrated a faint red fluorescent signal from donor cells (***B***). Scale bar 500 μm.

The differences between rMSCs delivery and scaffold augmentation in regards to histology and mechanical properties warranted an investigation into the fate of the cells delivered by means of the electrospun biomimetic scaffold. Cell tracing was performed to help distinguish if the beneficial effects of MSCs were from direct contribution to the repair vs. indirect mechanisms. Tracing of the donor cells was accomplished *via* staining the rMSCs plasma membrane with the well-established PKH staining system [38] where the fluorescent signal is maintained for 100 days in red blood cells, however may be lost more rapidly in dividing cells. Of three animals euthanized at 6-weeks post-surgery, only one demonstrated an island of signal next to the scaffold as illustrated in **Fig 7**. None of the animals at 12-weeks demonstrated any cell signal. For the most, there was no significant presence of donor cells at the later time points (**Fig 7**). Similarly, rat rotator cuff defects treated with MSCs showed a significant decrease in donor cells from 2 to 4 weeks [51], as well as in other tendons/species [52–54].

## Discussion

A pattern of repair (suture alone) has been established in the literature for rat supraspinatus tear model. During the initial repair of the supraspinatus, there is loss of bone to tendon transition, [55] disorganized collagen fiber orientation and increased concentration of enlarged, round, random oriented tenocytes [45] and a mostly Col III rich matrix [56]. With time the cells and fibers orient in the direction of tensile load or strain, cellularity decreases and the transition zone at tendon insertion undergoes remodeling [55] dependent on the degree of regeneration. As the ECM at the repair site remodels, the ratio of Col I: Col III expression increases with time as the initial scar tissue composed largely of Col III is replaced [56]. With this pattern in mind, RC augmentation using the ECM mimetic scaffold delivering rMSCs demonstrated a greater degree of healing compared to the other experimental groups as evidenced by Mason Trichrome staining (**Figs 2 and 3**). Further, the ratio of Col I: Col III within the repair tissue is similar to that of intact RC tendon as would be expected for a significantly improved healing response (**Fig 4**).

Suture alone repair and scaffold augmentation groups in the present work (**Fig 5**) demonstrated less birefringence transmission from the tendon body compared to matrix/rMSCs repair and intact tendon. As Col I is normally found in organized fibrous tissue, it is not surprising that the trend observed for Col I: Col III ratio is mirrored by quantification of tissue birefringence. Similar observations were found by Kovacevic *et*. *al*. in which TGF-ß, when applied to supraspinatus repair, achieved both greater Col I: Col III staining ratio and more advanced tendon healing according to morphology and tissue birefringence over suture repair alone [40]. To summarize, the histological effect of cell delivery on RC repair is two-fold, first, it results in a transition zone at the bone-tendon insertion similar to intact tendon tissue. Secondly, the delivery of cells using the biomimetic scaffold leads to a more mature or tendon-like connective tissue underlying the scaffold as compared to the scaffold alone repair.

As shown in **Fig 6**, mechanical characteristics of the intact tendon in regards to modulus, ultimate load, and ultimate stress are in line with previous reports [45–48], attesting to the validity of the testing system. Cross-sectional area, ultimate stress, and modulus of the repaired RC tendon in the experimental groups deviated sharply from intact tendon as expected from studies of rat supraspinatus repair [48] with and without a scaffold or biologic factors [49]. The composition and design of the hybrid nanofiber scaffold envisions a gradual transfer of mechanical loading onto the repaired tendon tissue as the biomaterial degrades over time. Augmentation of the RC tendon tear is expected to significantly reduce re-tear rates during the initial healing period, and continue into the later healing phases as the biomaterial degrades and repair tissue remodels both in ECM alignment and composition leading to superior mechanical properties.

Between the experimental groups there existed meaningful mechanical differences, mainly that matrix augmentation without cells and suture alone repair performed similarly (**Fig 6**), while rMSCs delivery resulted in improved mechanical properties and tendon dimensions. Other studies of augmented repair have shown that the incorporation of matrix alone does not suffice to improve rat supraspinatus repair tissue mechanical characteristics [40]. Tendon repair using mesenchymal stem cell-seeded collagen gel implants resulted in increased load properties and collagen fiber alignment within the repair tissue in both a rabbit Achilles and patellar tendon injury model [57,58]. Similarly, the repair of infraspinatus tendon defects in rabbits using polyglycolic acid sheet seeded with MSCs achieved regeneration of the tendon-bone insertion and exhibited a gradual increase in mechanical properties [59]. The improved morphology and the decreased cross-sectional area of the biomimetic matrix/rMSCs delivery group suggest the presence of a more mature collagen tissue in the tendon body, an observation encountered in rat supraspinatus tendon repairs supplemented with growth factor secreting MSCs [60]. This reinforcing effect of biologics and scaffolding was observed in improved RC augmentation and tendon-bone insertion using calcium phosphate matrices in which the addition of TGF-β increased ultimate load and stress at 4-weeks post-surgery [40]. Given the modest effect of scaffold only augmentation on mechanical properties, a likely route would be to bypass the use of a delivery scaffold and simply apply MSCs to the repair of massive RC tears. Interestingly, the application of rMSCs to rotator cuff suture repair does not result in an increase in mechanical properties of the repair despite the cells surviving and remaining in the tissue for more than 4 weeks [51]. The overexpression of scleraxis (Scx) [61] or bone morphogenetic protein-13 (BMP-13) [60] leads to an improvement in mechanical properties, with ultimate stress and load at 4 weeks approaching that of the biomimetic matrix/rMSCs delivery group at 6 weeks.

The measured mechanical properties of the experimental groups correlate with the histological findings. Several studies have presented a strong correlation between histology of the repair tissue and mechanical properties, with greater tendon maturity resulting in a stronger tendon [42,43,61–64]. This correlation extends to improved mechanical characteristics and increased Col I: Col III area in the repair groups of the present work (**Fig 4**) as well as that by Kovacevic *et al*. [40]. Increased birefringence transmission (**Fig 5**) and the corresponding improvement in functional properties of the biomimetic scaffold with rMSCs delivery versus scaffold augmentation alone is similar to other published observations [41,42]. Collectively, RC repair using biomimetic scaffold delivering rMSCs helped restore tendon morphology leading to repair tissue with significantly improved structural integrity that is more similar to intact tendon tissue.

The hypovascular nature of the tendon tissue lends to a poor nutrient supply which may limit the viability of donor cells resulting in cell death or migration. Collectively, in the aforementioned studies, the absence of donor MSCs in the shoulder tissue indicates that the implanted cells are acting in a trophic manner [65] to improve tendon healing, with the scaffold anchoring this effect to the regenerating tissue. Trophic effects of MSCs are believed to occur through several mechanisms one of which is by paracrine/autocrine modulation through GF secretion [66]. Several GFs involved in tendon development/repair such as TGF-β, fibroblast growth factor-2 (FGF-2), platelet-derived growth factor (PDGF), and vascular endothelial growth factor (VEGF) are secreted by MSCs. In addition, there is strong evidence for ECM deposition influenced by donor cells and thereby modulating the repair of tendon tissue. For example, embryonic stem cell-derived MSCs implanted into rat patellar defects secrete chemokines and recruit local host cells, leading to ECM and growth factor secretion which guide the differentiation of host cells despite the lack of donor cell incorporation [53]. Exogenous MSCs may also play a role in modifying the inflammatory environment during repair [67]. Healing during RC repair is atypical and incomplete [68], thus the presence of MSCs at initial repair may attenuate the inflammatory process to result in greater resolution of tendon regeneration.

An alternative trophic mechanism is that the implanted MSCs are guiding stem cell homing (reviewed by Sohni and Verfaillie [69]). There is growing evidence that stem cell homing helps determine the degree to which tissues can regenerate by the attenuation or strengthening of specific repair processes. MSCs deposit/secrete chemotactic factors which influence a variety of cell-types including endothelial cells, macrophages [70], fibroblasts [71], and other MSCs (autotaxis). The work by Shin and Perterson demonstrates that implanted MSCs participate in the homing of other stem cells during repair in a wound window mouse model [72]. The grafting of MSCs was found to accelerate healing in wild-type mice and normalize healing in diabetic mice compared to ungrafted control wound windows. Labeling of host cell prior to MSCs grafting indicated that those MSCs found in the wound bed were not expanded donor cells, but recruited host stem cells. This effect has also been observed in porcine myocardial infarction (MI) repair models [73]. MSCs implanted into the myocardium post-infarction led the recruitment of host cardiac stem cells from the surrounding tissue and improved tissue regeneration. Interestingly, MSC-conditioned media does not have this effect, which suggests that intact MSCs are required to interact with the regenerative environment in order to guide the reparative and stem cell homing response. With this in mind, the biomimetic scaffold in the present work delivers MSCs and may function to maintain them within the local microenvironment or niche to respond to the repair appropriately and mount a trophic response. Regardless, donor MSC allows for the recruitment of host stem cells that can directly contribute to tissue regeneration and/or act via trophic mechanisms to bolster repair.

## Conclusions

In conclusion, the present work is the first to combine an electrospun scaffold mimicking the tissue microenvironment and MSC delivery using the rat RC augmentation model. The hybrid fibrous construct consisting of an electrospun core of PCL fibers and surface modified with PNEA-mPh presented a modest effect on tendon biomechanics, resulting in regenerated tendon morphology similar to suture repair controls. However, matrix augmentation and the delivery of rMSCs increased biomechanics resulting in tissue morphology that closely resembled intact tendon indicating accelerated tendon remodeling as compared to other repair groups. Cell tracking presented only traces of donor cells at 6-weeks, which suggests that the observed therapeutic effect is based on paracrine release/deposition of growth factors, chemotactic molecules, and/or immune modulators. Regardless of the mechanism, the acute improvement in healing provides evidence that we can clinically intervene immediately after the injury to decrease the amount of initial pathologic damage and/or enhance the rate of tendon remodeling. The next step is to apply electrospun nanofiber matrices to deliver MSCs in studies involving large animal models of RC repair. Using rabbit or sheep shoulders will help determine the biomechanical contribution of cell seeding at a higher resolution while utilizing suture anchors and ties similar to that employed in a human RC repair.
